# Torque Teno Viral Plasma Load for Immunologic Monitoring in Solid Organ Transplantation: One Step Further

**DOI:** 10.1097/TP.0000000000004817

**Published:** 2023-10-06

**Authors:** Frederik Haupenthal, Gregor Bond

**Affiliations:** 1 Division of Nephrology and Dialysis, Department of Medicine III, Medical University of Vienna, Vienna, Austria.

The introduction of calcineurin inhibitor (CNI)–based immunosuppression in the 1980s significantly reduced acute graft rejection after solid organ transplantation (SOT). During the past 40 y, this immunosuppressive regimen has remained largely unchanged, and it is unlikely that new drugs will drastically alter routine care in the next decades. However, SOT still faces 2 major challenges: chronic graft rejection and opportunistic infections, both of which result from inappropriate dosed immunosuppression. Because xenotransplantation and tolerance-inducing strategies are not yet practical for broad clinical use, personalized medicine focusing on individual optimization of the existing immunosuppressive regimen becomes a timely strategy.

In this respect, the quantification of the Torque Teno virus (TTV) has been introduced as an immunometer to assess the individual immunocompetence.^1,2^ Almost all healthy individuals and SOT recipients are infected with 1 to 22 species of these nonpathogen *Anelloviruses*. TTV plasma load is detected by means of real-time polymerase chain reaction, and since 2021, a commercial assay with Conformité Européenne certification is available for clinical use. Plasma TTV load is associated with the risk of graft rejection and infection in kidney, lung, and liver transplant patients and it is hypothesized that TTV load indirectly reflects the immunocompetence of its host: a high TTV load indicates insufficient viral control because of intense immunosuppression and a low TTV load indicates a competent immune system because of low immunosuppression. Currently, 3 randomized controlled trials are recruiting over 500 kidney and lung transplant recipients in 7 European countries to test the value of TTV-guided immunosuppression with results expected from 2024 (TTVguideIT, VIGILung, and TAOIST).^3,4^ Until then, TTV load cut-off values for risk stratification of graft rejection and infections based on observational studies might be applied for routine post-transplant care.^1,5^

The association between TTV load and the type and dosage of immunosuppression has been established. However, limited information exists about the kinetics of TTV load following adjustments in immunosuppressive dosages. Understanding the magnitude and timing of TTV load changes is of paramount importance. Despite interventional studies concentrating on CNI adjustments, few researchers have explored the effects of altering antimetabolite dosages. In this context, the study conducted by Benning et al^6^ from Heidelberg, Germany, is highly appreciated. Their study offers valuable clinical insights into the dynamics of TTV load alterations consequent to modifications in mycophenolic acid (MPA) dosing.^6^ The study not only reaffirms but also builds on the prior findings of Regele et al,^7^ who observed a decrease in TTV load after a 2-wk pause in MPA administration. The current post hoc analysis of a prospective, observational single-center study by Benning et al^6^ quantified TTV load before and after 1 mo’s MPA pause in 43 immunologic low-risk kidney transplant recipients. The intention behind this MPA pause was to enhance the response to the severe acute respiratory syndrome coronavirus 2 (SARS-CoV-2) messenger RNA (mRNA) vaccine. The study’s participants had an average age of 58 y, with 33% being female. They were more than 4 y post-transplant and were undergoing CNI-based immunosuppression (mainly tacrolimus), alongside an average daily MPA dosage of 1.5 g. Commercial Conformité Européenne–marked real-time polymerase chain reaction was used to analyze TTV plasma load. TTV load declined from a median of 4.0 log copies/mL (c/mL) to 3.7 log c/mL after 1 mo’s MPA pause. Two months after reinitiation of MPA, TTV load increased to 4.3 log c/mL once again.

How should we interpret these findings in the context of existing literature? Both Benning et al^6^ and Regele et al^7^ reported a 50% decrease in TTV load following 1 mo and 2 wk of MPA pause, respectively. Both studies identified a reduction in TTV load between 1 mo and 6 wk after the MPA pause. It is important to note that TTV load typically reaches its peak around the third month after the initiation of immunosuppression post-transplant, suggesting that it might take up to 3 mo for TTV load to stabilize after modifications to immunosuppression. Consequently, it remains uncertain whether the observed reduction in TTV load truly signifies the nadir following the MPA pause. In the study by Regele et al,^7^ TTV load remained low 1 mo after reintroduction of MPA and no further measurements were available. In the study by Benning et al,^6^ TTV load rebounded to baseline values 2 mo after the reintroduction of MPA.

In conclusion, the existing body of literature implies that a pause in MPA lasting between 2 and 4 wk results in a significant reduction in TTV load. Furthermore, a state of equilibrium in TTV load is typically achieved 6 to 8 wk after modifications to MPA dosage. Taking these findings into consideration, we recommend that monitoring TTV response subsequent to alterations in immunosuppression should extend for a minimum of 2 mo postadaptation. However, more comprehensive investigations featuring more granular measurements of TTV load spanning up to 3 mo after MPA adjustments are necessary to pinpoint the optimal timing for assessing TTV load response accurately. Additionally, the impact of a 50% reduction in MPA dosage—a current practice during opportunistic infections—on TTV kinetics remains a subject that requires examination. Above all, studies investigating TTV kinetics subsequent to adaptations in CNI dosage are still indispensable for a comprehensive understanding.

Another facet of the research conducted by Benning et al,^6^ which merits further discussion, is their achievement in enhancing the response to SARS-CoV-2 mRNA vaccination through MPA pause in relation to TTV load. To date, conflicting outcomes have been documented about strategies aimed at optimizing SARS-CoV-2 vaccination response via MPA pause. In the study performed by Benning et al,^6^ a 1-mo pause in MPA usage resulted in a reduction of TTV load to 3.7 log c/mL and facilitated an increased vaccination response. Conversely, a 2-wk pause in MPA administration, as explored in the study conducted by Regele et al,^7^ led to a TTV load reduction only to 3.9 log c/mL (about one-third higher) and did not yield heightened vaccination response rates. In this context, investigating the potential of a TTV-guided MPA pause to enhance vaccination response within a randomized controlled framework appears to be a promising avenue for exploration.

Finally, in accordance with previously published studies, Benning et al^6^ described a correlation between TTV load and SARS-CoV-2–specific anti-S1 IgG titers in patients with MPA pause. Other researchers have also demonstrated an association between TTV load and the response to SARS-CoV-2 mRNA vaccination.^7–9^ Seroconversion rates were acceptable among kidney transplant recipients exhibiting a TTV load below 2.6 log c/mL, whereas patients with a TTV load surpassing 4.6 log c/mL displayed low rates of seroconversion.
